# ROAD2H: Development and evaluation of an open‐source explainable artificial intelligence approach for managing co‐morbidity and clinical guidelines

**DOI:** 10.1002/lrh2.10391

**Published:** 2023-09-12

**Authors:** Jesús Domínguez, Denys Prociuk, Branko Marović, Kristijonas Čyras, Oana Cocarascu, Francis Ruiz, Ella Mi, Emma Mi, Christian Ramtale, Antonio Rago, Ara Darzi, Francesca Toni, Vasa Curcin, Brendan Delaney

**Affiliations:** ^1^ Department of Population Health Sciences King's College London London UK; ^2^ Imperial College London London UK; ^3^ University of Belgrade Belgrade Serbia; ^4^ Department of Informatics King's College London London UK; ^5^ London School of Hygiene and Tropical Medicine London UK; ^6^ University of Oxford Oxford UK

**Keywords:** argumentation, CDS hooks, clinical decision support systems, co‐morbidity, FHIR, Transition‐based Medical Recommendation model

## Abstract

**Introduction:**

Clinical decision support (CDS) systems (CDSSs) that integrate clinical guidelines need to reflect real‐world co‐morbidity. In patient‐specific clinical contexts, transparent recommendations that allow for contraindications and other conflicts arising from co‐morbidity are a requirement. In this work, we develop and evaluate a non‐proprietary, standards‐based approach to the deployment of computable guidelines with explainable argumentation, integrated with a commercial electronic health record (EHR) system in Serbia, a middle‐income country in West Balkans.

**Methods:**

We used an ontological framework, the Transition‐based Medical Recommendation (TMR) model, to represent, and reason about, guideline concepts, and chose the 2017 International global initiative for chronic obstructive lung disease (GOLD) guideline and a Serbian hospital as the deployment and evaluation site, respectively. To mitigate potential guideline conflicts, we used a TMR‐based implementation of the Assumptions‐Based Argumentation framework extended with preferences and Goals (ABA+G). Remote EHR integration of computable guidelines was via a microservice architecture based on HL7 FHIR and CDS Hooks. A prototype integration was developed to manage chronic obstructive pulmonary disease (COPD) with comorbid cardiovascular or chronic kidney diseases, and a mixed‐methods evaluation was conducted with 20 simulated cases and five pulmonologists.

**Results:**

Pulmonologists agreed 97% of the time with the GOLD‐based COPD symptom severity assessment assigned to each patient by the CDSS, and 98% of the time with one of the proposed COPD care plans. Comments were favourable on the principles of explainable argumentation; inclusion of additional co‐morbidities was suggested in the future along with customisation of the level of explanation with expertise.

**Conclusion:**

An ontological model provided a flexible means of providing argumentation and explainable artificial intelligence for a long‐term condition. Extension to other guidelines and multiple co‐morbidities is needed to test the approach further.

**Abbreviations**
ABA+G: assumptions‐based argumentation framework extended with preferences and goals
AI: artificial intelligence
ALS: airflow limitation severity
API: application programming interface
CAT: COPD assessment test
CB: causation belief
CDS: clinical decision support
CDSS: clinical decision support system
CG: computable guideline
CKD: chronic kidney disease
COPD: chronic obstructive pulmonary disease
COVID‐19: coronavirus disease 2019
CQL: Cassandra query language
CVD: cardiovascular disease
EHR: electronic health record
EPSRC: engineering and physical sciences research council
FHIR: fast health interoperability resources
GOLD: Global Initiative for Chronic Obstructive Lung Disease
GUI: graphical user interface
HL7: health level 7
ICS: inhaled corticosteroids
ID: identity
JSON: Javascript order notation
LABA: long‐acting beta‐agonist
LMIC: low‐ and middle‐income countries
mMRC: modified medical research council
NICE: National Institute for Health and Care Excellence
NIHR: National Institute for Health and Care Research
OA: osteoarthritis
RDF: resource description framework
ROAD2H: resource optimisation, argumentation, decision support and knowledge transfer to create value via learning health systems
SABA: short‐acting beta‐agonist
SAMA: short‐acting muscarinic‐antagonist
SMART: standards‐based, machine‐readable, adaptive, requirements‐based, and testable
SNOMED CT: systematized nomenclature of medicine clinical terms
SPARQL: SPARQL protocol and RDF language
SQL: structured query language
SWI‐Prolog: Sociaal‐Wetenschappelijke Informatica—programming in logic
TMR: Transition‐based Medical Recommendation
URI: uniform resource identifier
WHO: World Health Organization

## 1 BACKGROUND

Increasingly, developers of clinical guidelines, such as the World Health Organization (WHO) and the National Institute for Health and Care Excellence (NICE)^1^ in the United Kingdom, have sought to implement aspects of guidance via encouraging the creation of computational algorithms, often proprietary, within electronic health records (EHR). However, multimorbidity, defined as the coexistence of two or more chronic medical conditions in an individual patient,^2^ is common in the real world and presents a multitude of competing priorities, potential contraindications, and guideline exceptions to the clinician. Although studies have shown clinical decision support (CDS) improves clinicians' adherence to clinical and operational guidelines for medication, prevention, and treatment,^3^, ^4^, ^5^, ^6^ problems with ‘alert fatigue’, confusion and contradiction between different CDS alerts can represent a threat to patient safety.^7^ ​​Computable guidelines (CGs), machine‐interpretable versions of guidelines, have the potential to alleviate some of this burden on the clinician by using ‘argumentation’, that is, defining the ‘best’ option from a series of logical statements.^8^ However, in low‐ and middle‐income countries (LMIC), the resources required for proprietary EHR‐integrated CDS systems (CDSSs), localization and maintenance are often not available.^9^ In contrast, open source code can be written to extract data and trigger a rule externally. For example, using both the FHIR API, a global standard to promote data‐level interoperability among disparate EHRs, and CDS Hooks,^10^ a specification for standardising the seamless integration of external services in EHRs. Growing functionality in CDS can be represented as multiple interacting models and ontologies as written rules are replaced by computable ones,^11^ allowing, for example, a model of the patient's current clinical state to present data to a model of appropriate guideline statements, applied to derive a care recommendation.

To meet the challenge of co‐morbidity, a model of clinical reasoning between conflicting statements is required. Argumentation models amount to automated systems that emulate human reasoning,^12^ positioning arguments and counterarguments for a given issue to find the ‘winning’ arguments. Argumentation is a good fit for modelling patient‐centric reasoning with multiple guidelines where interacting recommendations and alerts give rise to conflicts, and the context of a patient brings in various conditions, goals, and preferences.^13^

The EPSRC‐funded ROAD2H project aimed to develop and evaluate a representative CDSS for embedding and enacting the internationally accepted Global Initiative for Chronic Obstructive Lung Disease^14^ (GOLD) guideline, illustrating the role of argumentation‐based techniques in presenting conflict‐safe care plan proposals to clinicians, and integrating the system with a modern standards‐based commercial EHRs in a middle income country (Heliant,^15^ the largest healthcare information systems provider in Serbia) using a combination of knowledge representation and interoperability standards.

## 2 METHODS

### 2.1 Clinical case study

We chose the management of chronic obstructive pulmonary disease (COPD) with cardiovascular (CVD) or chronic kidney disease (CKD) co‐morbidities as an exemplar. The GOLD guideline classifies the COPD symptom severity as a series of stages or groups, each with recommended treatments in a ‘step up’ fashion. In addition, some of the therapies are contraindicated in co‐morbidity, for example, beta agonist medications with angina.^14^ COPD is increasingly common in LMIC, so we aimed at designing a CDSS that integrates both COPD symptom severity assessment and treatment planning with the workflow of pulmonologists in the EHR. Requirements were to identify potential treatment conflicts and suggest alternative care plans.

### 2.2 The Transition‐based Medical Recommendation (TMR) model

To reason about potential interactions among, or within, guidelines, we adopted the TMR model,^3^, ^16^ a formalism to represent guidelines and detect conflicts using semantic web technologies and logic rules. TMR‐represented recommendations (see Table 1) comprise a care action and its causation belief, that is, the expected effect on the measured property the care action affects. Care actions promote transitions which comprise the initial (clinical) state and the expected state of the affected measured property. Care action effects potentially increase or decrease the value of the measured property associated with a recommendation. There are implementations of TMR for prototyping guideline representation (using the RDF model for guideline representation and SPARQL^17^ as its query language) and interactions theory (using the logic‐based programming language SWI‐Prolog); however, neither implementation offers enactment nor automated merging of CG statements. Like other multimorbidity‐oriented formalisms,^4^, ^5^ TMR currently lacks the reasoning capabilities to resolve the identified interactions and does not consider patient‐specific conditions, goals, or treatment and lifestyle preferences.^6^ The computational argumentation formalism described next aims to integrate all these elements to provide automated reasoning with interacting TMR‐based CGs, considering the patient's context and preferences.

**TABLE 1:** Recommendations based on GOLD guideline, represented as TMR knowledge.

Rec ID	English representation	Care action	CB ID	Measured property	Initial state	Expected state after care action application	Expected degree of change on measured property
*R*_sama_	Recommend administering SAMA bronchodilator to COPD patients with mild ALS	Administer SAMA bronchodilator	CB1	ALS	Mild ALS	Mild ALS	Maintain
*R*_saba_	Recommend administering SAMA bronchodilator to COPD patients with mild ALS	Administer SABA bronchodilator	CB2	ALS	Mild ALS	Mild ALS	Maintain
*R*_beta_	COPD patients with co‐morbid cardiovascular disease should be aware that beta‐agonists bronchodilators could increase the risk of cardiac rhythm disturbances	Administer beta‐agonist bronchodilator	CB3	At risk of cardiac rhythm disturbances	Low risk	High risk	Increase
*R*_jab_	Recommend administering pneumococcal vaccine to patients over 64 years of age	Administer pneumococcal vaccine	CB4	At risk of pneumonia	High risk	Low risk	Decrease

Abbreviations: ALS, airflow limitation severity; CB, causation belief; Rec, recommendation; SABA, short‐acting beta‐agonist; SAMA, short‐acting muscarinic antagonist.

### 2.3 Explainable argumentation

**FIGURE 1:**
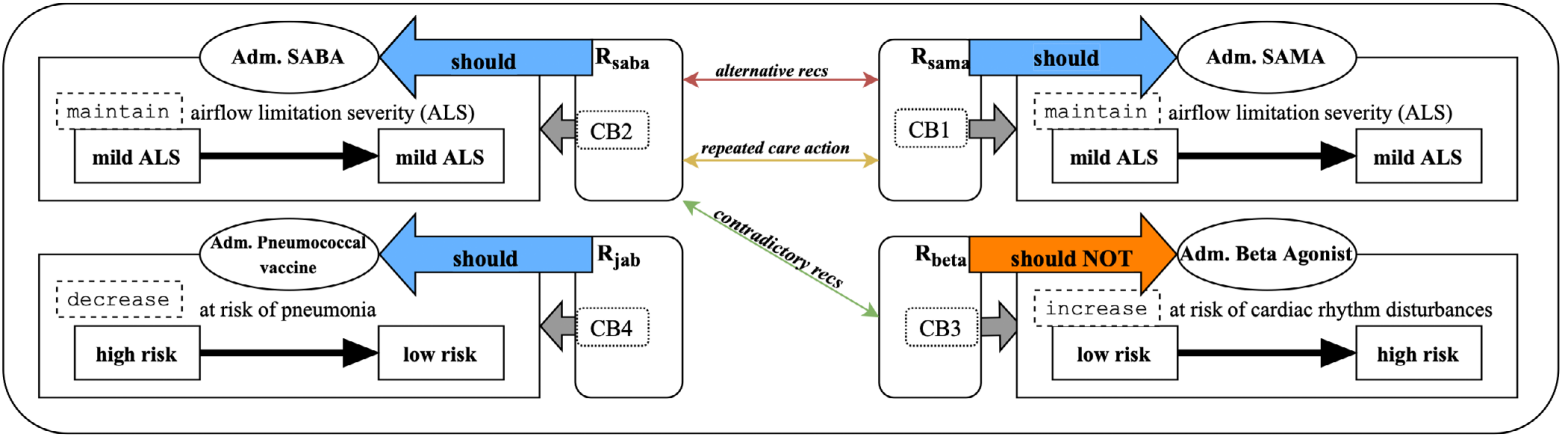
Graphical representation of the recommendations in Table 1, and their identified potential interactions when administered together. Adm. stands for administer, recs for recommendations, CB for causation belief (there are exclusively one per recommendation for the iteration of the TMR model applied in this project), SABA (SAMA) stands for short‐acting beta‐agonist (muscarinic antagonist) bronchodilator.

To reason over the guideline conflicts, we made use of the *Assumption‐Based Argumentation with Preferences and Goals* ^13^ (ABA+G) technique, which represents knowledge using a formal (logical) language, rules, and defeasible assumptions. Rules and assumptions allow for a transparent and interpretable representation of the TMR concepts, particularly recommendations and their components, as illustrated in Figure 1, where interactions among recommendations can likewise be captured via rules. For example, and following Figure 1, the contradictory interaction between recommendations *R*_*saba*_ and *R*_*beta*_ is expressed via two rules that assume *R*_*saba*_ leads to an objection against *R*_*beta*_, and vice versa. Moreover, since *R*_*sama*_ is identified as an alternative recommendation to *R*_*saba*_, then acceptance of *R*_*beta*_ leads to the acceptance of *R*_*sama*_ by application of a rule which assumes both that *R*_*beta*_ and *R*_*Saba*_ object each other and that *R*_*beta*_ and *R*_*sama*_ do not object each other.

Explainability, as in not only providing explanations accompanying reasoning outcomes, but also the overall transparency in knowledge representation and reasoning mechanisms, is a fundamental requirement of CDSS.^18^ Argumentation is itself an explainable reasoning paradigm,^19^ and it has also given rise to explanations in various settings, including AI.^20^, ^21^, ^22^ ABA+G natively affords means to explain its reasoning outcomes. Specifically, ABA+G yields explanations of the reasoning trace, its actions and expected effects. Explanations summarise the considered interactions and preferences, and accompany each proposed set of recommendations (eg, Table 2). A TMR‐based implementation of ABA+G (hereinafter the conflict resolution service) was utilized in this project.^23^

**TABLE 2:** Response example by the conflict resolution service from Figure 2 using the TMR‐based knowledge from Figure 1.

Rec ID reference	Generated explanation	Alternative recs	Repeated care action	Contradictory recs	Extensions
*R*_sama_	administration of SAMA *has* positive contribution *on* airflow limitation severity *to* maintain *from* mild airflow limitation severity *to* mild airflow limitation severity	*R*_saba_ (+)	*R*_saba_ (+)	··	ext1
*R*_saba_	administration of SABA *has* positive contribution *on* airflow limitation severity *to* maintain *from* mild airflow limitation severity *to* mild airflow limitation severity	*R*_sama_ (−)	*R*_sama_ (−)	*R*_beta_	ext2
*R*_beta_	administration of beta agonist *has* negative contribution *on* at risk of cardiac rhythm disturbances *to* increase *from* low risk of cardiac rhythm disturbances *to* high risk of cardiac rhythm disturbances	··	··	*R*_sama_	ext1
*R*_jab_	administration of pneumococcal vaccine *has* positive contribution *on* at risk of pneumonia *to* decrease *from* high risk of pneumonia *to* low risk of pneumonia	··	··	··	ext1; ext2

*Note*: The structured clinical knowledge from Table 1 is also part of the response but omitted here. The underlined words on column Generated explanation denote the fixed parts of the explanation template. Rec(s) stands for recommendation(s). Symbol + (−) stands for preferable (less preferable). Column Extensions denotes to which extension(s) belongs the recommendation.

### 2.4 Integration with heterogeneous EHR systems

**FIGURE 2:**
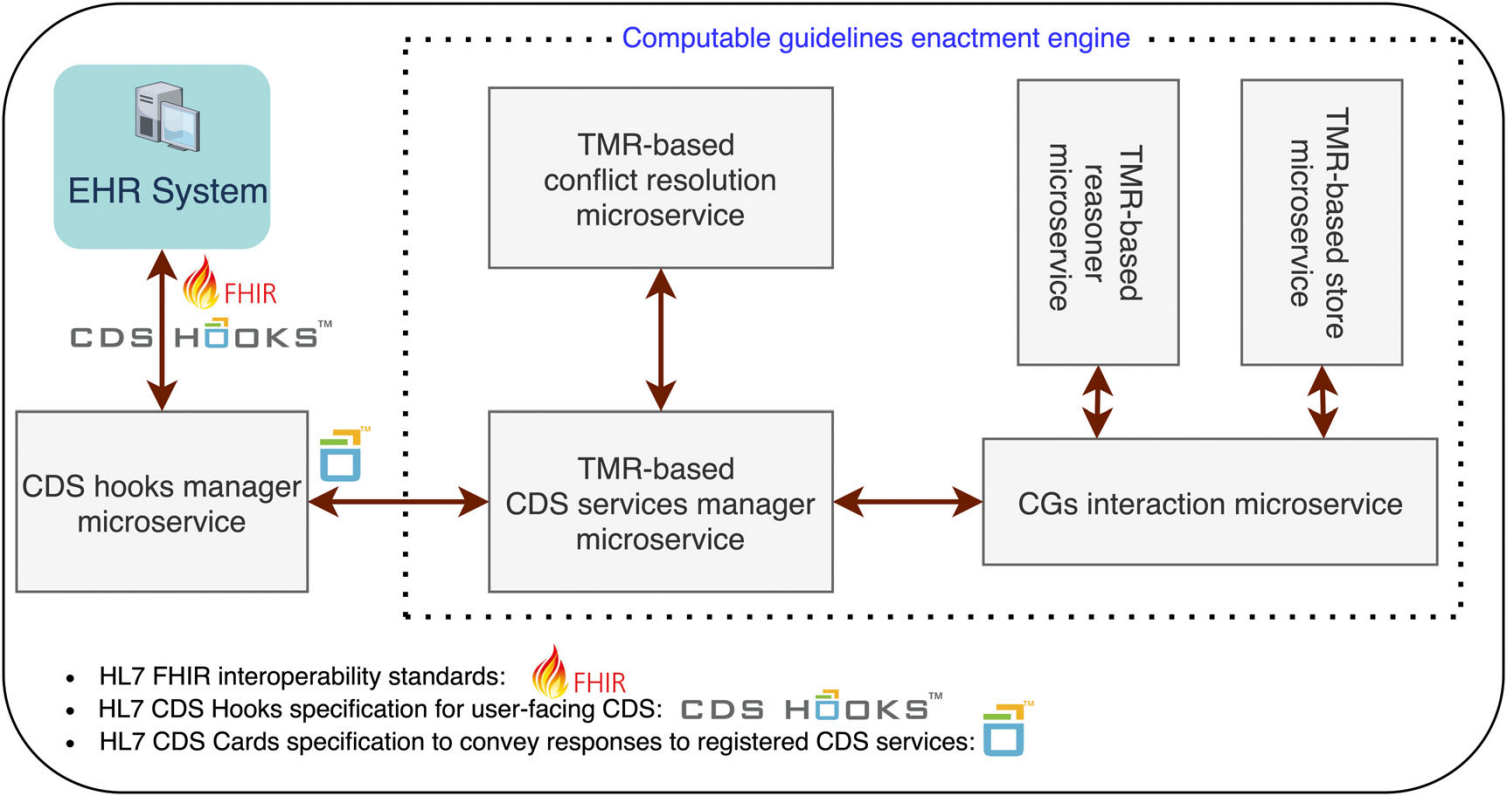
Architecture of the multipurpose, argumentation‐based TMR‐driven CDS system. The interoperability service is labelled as the CDS hooks manager microservice. The TMR‐based conflict resolution microservice implements ABA+G to handle TMR‐based knowledge. Similarly, the reasoner and CGs store microservices implement the TMR model and reasoning logic, the former, and a SPARQL server, the latter. Both managed by the CGs interaction microservice. The CDS services manager encapsulates the semantics of CDS services and the conversion of TM—based CIG‐specific knowledge into FHIR artifacts.

We designed a general‐purpose ontology‐based CDSS architecture based on open‐source and industry standards that consists of an interoperability service, and a CGs enactment architecture which includes ABA+G and extends on an existing CGs authoring microservice architecture.^24^ The interoperability service is common to any implementation and uses SNOMED CT and FHIR standards to exchange healthcare data with EHRs. In addition, CDSS‐subscribed EHRs invoke event‐specific CDS according to CDS hooks specification standards where notifications for CDS and their responses are delivered in the form of hooks and cards, respectively.^10^ Cards convey information determined by implementations of the CDSS architecture for specific CG formalisms and CDS events (eg, TMR and COPD treatment planning, respectively). Appendix A.2 furthers this description. A TMR‐based implementation of the generic CDSS architecture is illustrated in Figure 2 and discussed next.

To enable argumentation over TMR, we developed a suite of microservices (hereinafter the [TMR‐based] CDS framework) to enact, and personalise, TMR‐based CGs that includes the conflict resolution service and leverages a TMR‐based implementation of the CGs authoring microservice architecture.^24^ This implementation encapsulated the previously mentioned existing TMR technology to store, query, and reason about TMR‐based knowledge.^24^

**FIGURE 3:**
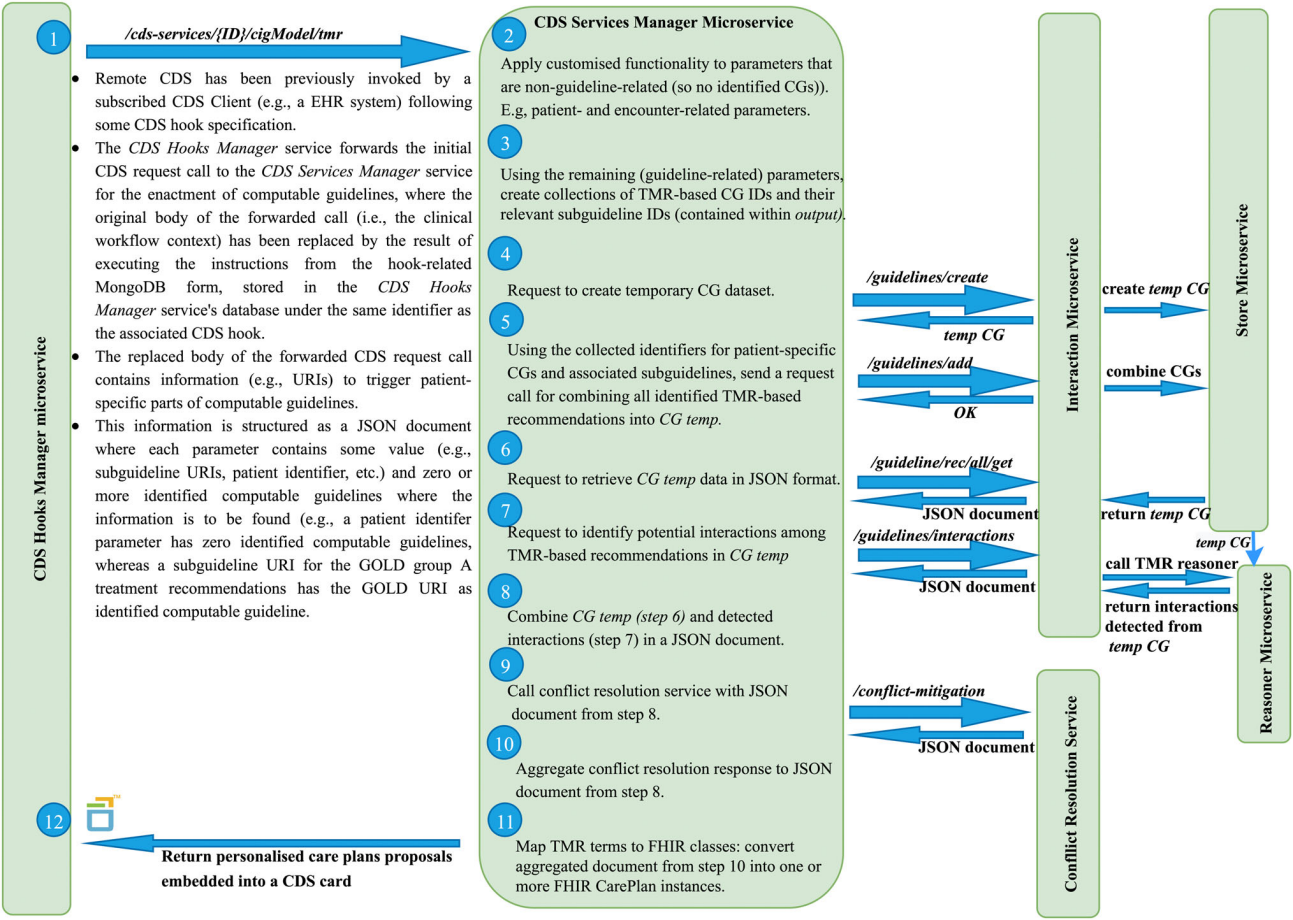
Workflow of the TMR‐based COPD framework. Modelled workflow of how CDS calls are handled by the CDS Services Manager microservice in the argumentation‐based, TMR‐driven CDS system.

Patient‐relevant parts of implemented TMR‐based CGs are triggered in the CDS framework by event‐specific data included in the context of the CDS call. Each triggered recommendation is identified by a pair of unique RDF URIs referencing the recommendation and its CG, and combined into a volatile dataset using SPARQL. TMR interaction detection rules are then applied to this dataset. The dataset and detected potential interactions are encoded in JSON alongside user‐defined preferences included in the hook's context, then forwarded to the conflict resolution service. The response of this service consists of a collection of ‘extensions’ originating from the dataset, where each extension comprises TMR recommendations aggregated by considering potential interactions within the extension and preferences. The *explainability* component then refactors the encoded knowledge from each recommendation into information for CDS in both computer‐interpretable and textual form. Finally, the response is embedded into a card by mapping extensions to FHIR carePlan types, resulting in personalized conflict‐free care plan proposals comprising triggered recommendations potentially from multiple CGs and which include mitigation information on potential interactions among triggered recommendations from the source dataset (eg, alternative recommendations distributed into distinct proposals). Figure 3 provides a modelled workflow of the system.

### 2.5 Evaluation framework

To evaluate our proposed CDS approach, we aimed to formalise key aspects of GOLD for stable COPD, including pharmacological, vaccination, physiotherapy, and smoking cessation therapies, plus drug‐disease warnings for CVD and CKD co‐morbidities. Appendix A.1 discusses the steps towards GOLD guideline formalisation using TMR. Subsequently, we defined hook specifications ‘copd‐assess’ (Table 3) and ‘copd‐careplan‐review’ for COPD management CDS (Table 4) and worked with Heliant to integrate the remote CDSS with their EHR via a graphical user interface (GUI) add‐on embedded in the EHR's COPD tab.

**TABLE 3:** Contextual information for hook ‘copd‐assess’.

Field	Optionality	Description
encounterId	REQUIRED	FHIR encounter.id of the current CDS process.
patientId	REQUIRED	FHIR patient.id of the current patient.
medication	OPTIONAL	COPD drug type, denoted by an internal Id, currently active. Omission of this resource suggests the patient has no previous COPD history prior to this encounter.
previousAssessment	OPTIONAL	FHIR bundle of observation instances representing, in SNOMED CT, COPD group, CAT and mMRC dyspnoea scale scores, and number of exacerbations as recorded on the previous COPD‐related encounter. Omission of the bundle resource suggests the patient has no previous COPD history prior to this encounter.
currentAssessment	REQUIRED	FHIR bundle of observation instances in ‘preliminary’ state representing CAT and mMRC dyspnoea scale scores, and number of exacerbations as measured at the current encounter.
asthma	OPTIONAL	FHIR condition instance denoting the presence of asthma in the patient's record.

*Note*: This CDS service collects patient data and COPD‐related measurements to assess the patient's COPD symptom severity, following GOLD's ABCD assessment algorithm, and to provide an ordered collection of COPD treatments, from most to least suitable to the patient, for each of the GOLD groups.

**TABLE 4:** Contextual information for hook ‘copd‐careplan’.

Field	Optionality	Description
encounterId	REQUIRED	FHIR encounter.id of the current CDS process
patientId	REQUIRED	FHIR patient.id of the current patient
birthDate	REQUIRED	Date of birth of the current patient. Supports decision on suggesting administering pneumococcal immunization to patients over 64 years of age
smokingStatus	REQUIRED	FHIR observation instance representing, via a SNOMED CT term, the patient as either a regular smoker or not.
co‐morbidities	OPTIONAL	FHIR bundle of condition instances representing, via SNOMED CT terms, active diagnoses in the record of the current patient.
immunizationStatus	REQUIRED	FHIR bundle of immunization instances denoting, via SNOMED CT terms, whether the patient has completed the annual influenza vaccine, or the pneumococcal vaccine.
copdAssessment	REQUIRED	FHIR bundle of observation instance and Medication bundle. The former represents, in SNOMED CT, the user‐selected COPD group. The latter represents, using internal Ids, the user‐selected COPD drug types requested for CDS.
cdsSuggestedTreatments	OPTIONAL	FHIR bundle of medication instances suggested by the COPD‐CDS system as a response to hook ‘copd‐assess’ for the current patient. The bundle is aggregated to the conflict resolution input document to provide alternatives to user‐selected COPD drug types when resolving potential conflicts among recommendations.

*Note*: This CDS service collects patient data from the EHR to propose one or more personalized COPD treatment management care plans.

We used simulated case vignettes to create dummy EHRs as access to real patients was prohibited by COVID‐19 restrictions. Two clinical authors (Ella Mi, Emma Mi) created 20 cases. Each case contained data as illustrated in Table 5. All GUI textual outputs were rendered in Serbian and validated by a Serbian clinician.

**TABLE 5:** Clinical case vignette of patient introduced in Figure 4.

Field	Value
Age	65
Sex	Male
Smoking status (SNOMED CT)	Ex‐smoker
GOLD	1
COPD drug type administered at last COPD assessment	None
Number of exacerbations in past 12 months at last COPD assessment	0
CAT score at last COPD assessment (SNOMED CT)	0
mMRC dyspnoea scale score at last COPD assessment (SNOMED CT)	0
COPD group assessed at last COPD assessment (SNOMED CT)	None
Number of exacerbations in past 12 months at current COPD assessment	0
CAT score at last COPD assessment (SNOMED CT)	6
mMRC dyspnoea scale score at last COPD assessment (SNOMED CT)	1
Asthma (SNOMED CT)	False
Influenza vaccine (SNOMED CT)	Completed
Pneumococcal vaccine (SNOMED CT)	Not‐done
Co‐morbidities (SNOMED CT)	Cardiovascular disease, hypertension

*Note*: Patient demographics, previous COPD clinical history and recorded number of exacerbations at current COPD assessment were populated into the EHR prior the evaluation. The remaining fields are entered by each clinician at evaluation time. SNOMED CT highlights that the condition or observation is recorded in the EHR using this clinical classification.

This was a mixed‐methods evaluation to analyse quantitatively the validity of the recommendations and qualitatively the clinicians' impressions of the approach. We recruited a purposive sample of five pulmonologists from Clinical Hospital Centre Zvezdara in Serbia to use the extended Heliant EHR with each of the 20 cases, providing 100 cases in total. First, we aimed to determine their agreement with the results of both CDS services for each clinical case. Second, after operating the system we interviewed each clinician using a structured questionnaire.

## 3 RESULTS

To evaluate our CDS approach for the management of patients with stable COPD, TMR representations of selected GOLD recommendations were defined and loaded into a dedicated SPARQL database. Similarly for the pair of hook context processing instructions described in Tables 6 and 7 (NoSQL database linked to the CDS hooks manager microservice), based on the specifications in Tables 3 and 4. This resulted in the specialisation of the TMR‐based CDSS for COPD symptom severity assessment and treatment planning decision‐making, hereinafter the COPD‐CDSS.

**TABLE 6:** Collection of JSON‐based documents applied to contextual data of CDS hook ‘copd‐assess’.

Document label	Application
copdSeverityAssessment	Returns a SNOMED CT‐based GOLD COPD group identifier for the active patient obtained by analysing their current COPD assessment results.
goldGroupA_treatmentPriorities	Assuming the active patient has a GOLD COPD group A symptoms severity, it returns an ordered list of suitable treatments by analysing their asthmatic status along with both previous and current COPD assessment results.
goldGroupB_treatmentPriorities	Assuming the active patient has a GOLD COPD group B symptoms severity, it returns an ordered list of suitable treatments by analysing their asthmatic status along with both previous and current COPD assessment results.
goldGroupC_treatmentPriorities	Assuming the active patient has a GOLD COPD group C symptoms severity, it returns an ordered list of suitable treatments by analysing their asthmatic status along with both previous and current COPD assessment results.
goldGroupD_treatmentPriorities	Assuming the active patient has a GOLD COPD group D symptoms severity, it returns an ordered list of suitable treatments by analysing their asthmatic status along with both previous and current COPD assessment results.
encounterID	Returns ID of this encounter.
patientID	Returns ID of this patient.
patientID	Returns ID of this patient.

*Note*: The collection is uploaded to the NoSQL database of the interoperability service when the CDS service is invoked by any subscribed EHR. Each document provides instructions to query, and manipulate, specific parts of the clinical workflow context data towards delivering CDS. Below, Column Document label identifies each instructions‐filled document in the collection. Column Application states the semantics of each document.

**TABLE 7:** Collection of JSON‐based documents applied to contextual data of CDS hook ‘copd‐careplan‐review’.

Document label	Application
co‐morbidities	Returns the TMR‐based CG ID for chronic kidney or cardiovascular diseases whenever a SNOMED CT‐based term found in the hook context is subsumed, or equals to, the SNOMED CT codes for chronic kidney or cardiovascular diseases or history of the diseases.
selected_copd_group	Returns the subguideline ID for the GOLD COPD treatment pathways associated with the selected COPD Group.
additional_selected_meds	Returns the COPD‐based subguideline ID(s) of additional COPD drug types that although are not officially part of the initial selection of GOLD COPD group drug types, are suitable to the active patient.
smoking_status	Returns the COPD‐based subguideline ID of a smoking cessation recommendation if the patient is identified, via SNOMED CT, as a smoker.
influenza_immunisation	Returns the COPD‐based subguideline ID of the influenza immunization recommendation if the patient has not completed the seasonal influenza vaccination.
pneumococcal_immunization	Returns the COPD‐based subguideline ID of the pneumococcal immunization recommendation if the patient's age is over 64 and no pneumococcal jab administration is recorded as completed.
selectedTreatmentPathways	Returns the list of COPD drug treatments selected by the user for requesting CDS.
alternativeTreatmentPathways	Returns the list of COPD drug treatments suggested by the COPD‐CDS system as a response to hook ‘copd‐assess’.
encounterID	Returns ID of this encounter.
patientID	Returns ID of this patient.

*Note*: The collection is uploaded to the NoSQL database of the interoperability service when the CDS service is invoked by any subscribed EHR. Each document provides instructions to query, and manipulate, specific parts of the clinical workflow context data towards delivering CDS. Below, Column Document label identifies each instructions‐filled document in the collection. Column Application states the semantics of each document.

**FIGURE 4:**
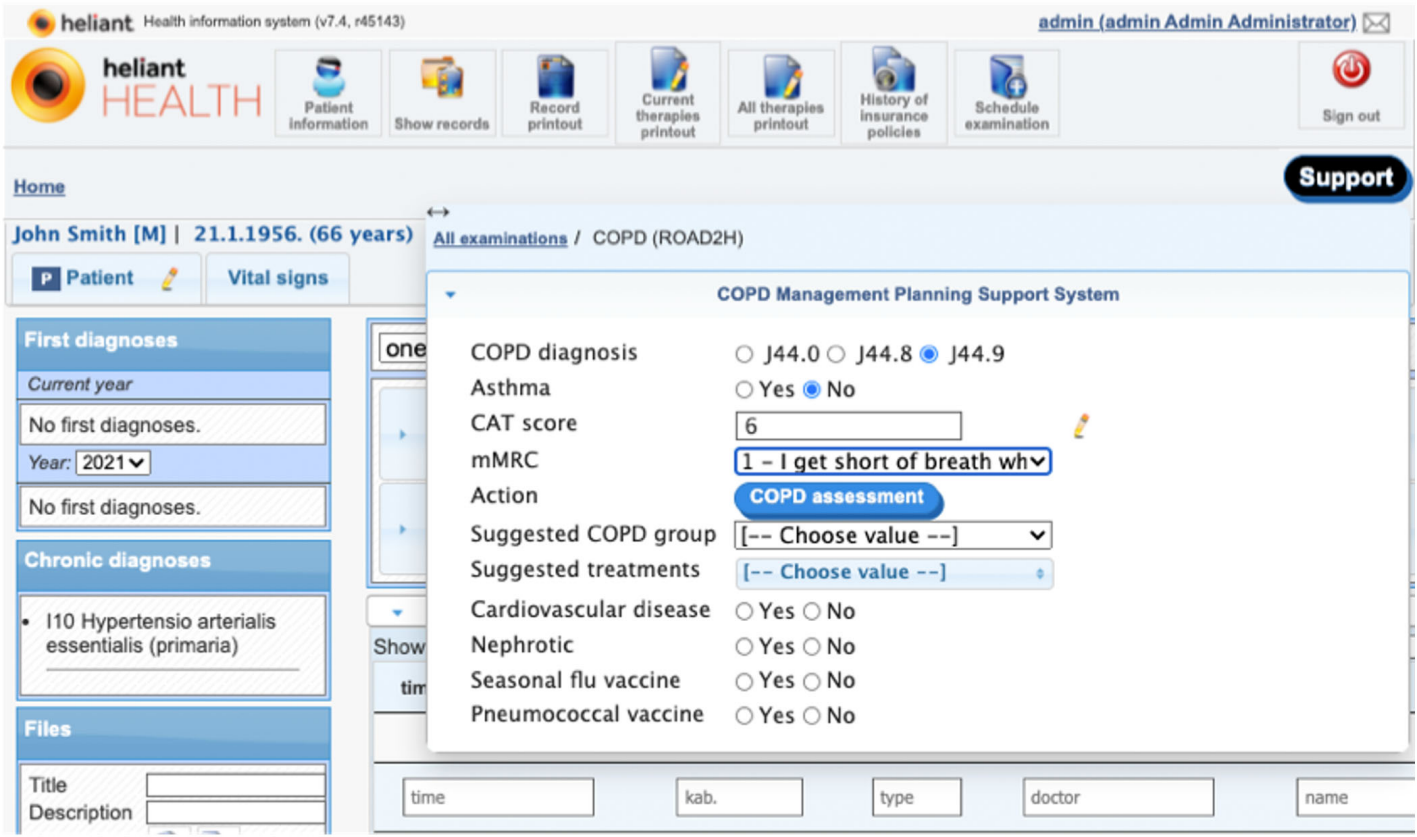
The COPD‐CDSS GUI presented on Heliant's EHR's COPD tab. The opened EHR belongs to a male patient with no previous COPD history. The COPD‐CDSS GUI is displaying the result of the patient's first COPD symptom severity assessment, entered by the pulmonologist. The ‘COPD assessment’ button triggers hook ‘copd‐assess’. The hook's response provides the content to fields ‘Suggested COPD group’ and ‘Suggested treatments’.

The COPD‐specialised GUI interacts with the COPD‐CDSS by collecting COPD‐related measurements and other relevant clinical details stated in both hooks' specifications, to aid with both clinical events. Each event invokes a CDS service, triggered their respective buttons in the GUI (see Figure 4). Using a GUI form to collect or modify input was a design choice made by Heliant that benefitted the pulmonologists when evaluating the COPD‐CDSS and was preferred by Heliant over the direct collection of relevant EHR data, which was perceived as less transparent and insufficiently interactive.

**FIGURE 5:**
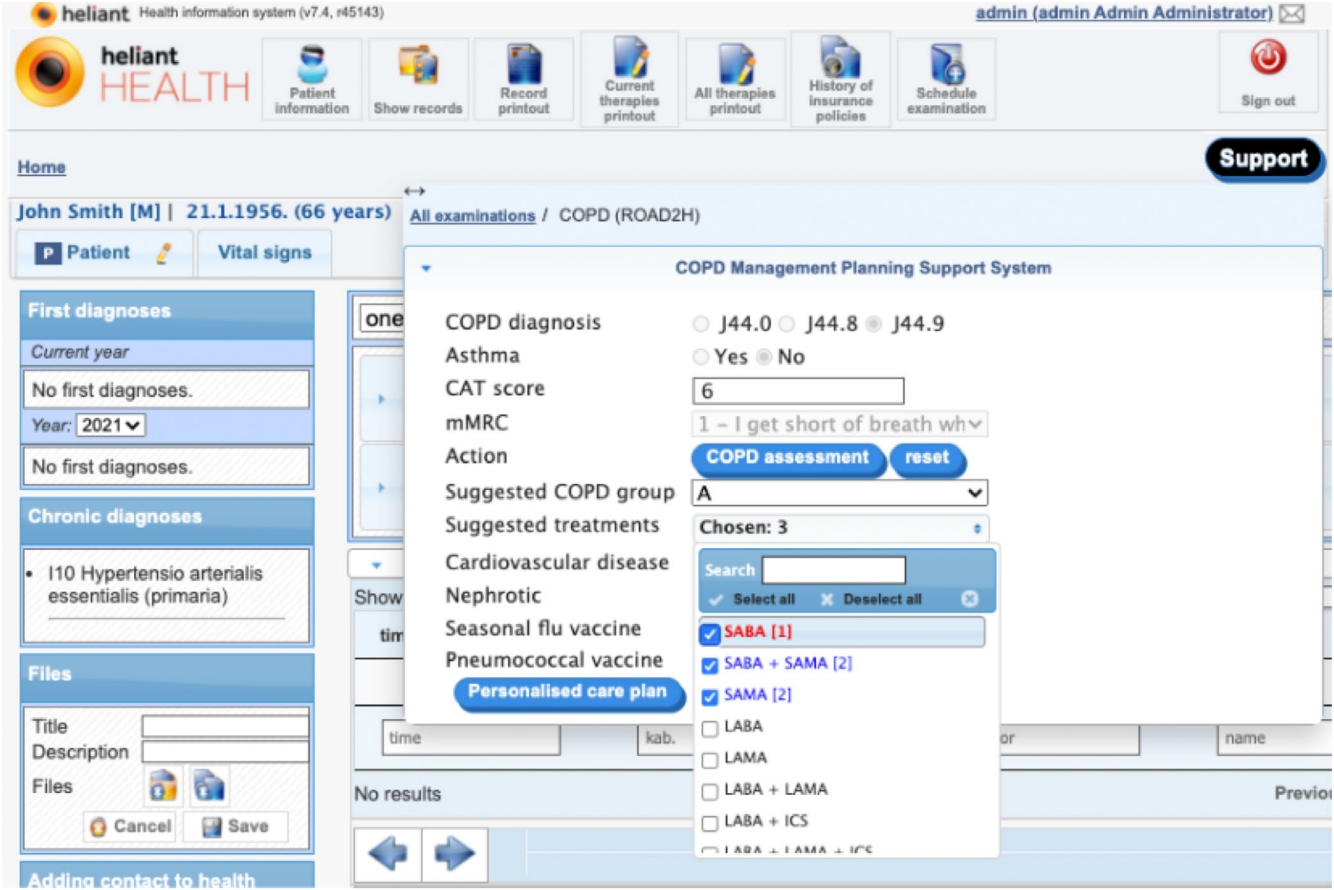
COPD severity assessment response as invoked by the CDS hook ‘copd‐assess’ in the COPD‐CDSS for the patient introduced in Figure 4.

Figure 5 shows the clinical contents of the card responding to hook ‘copd‐assess’ integrated with the GUI, that is, the personalized GOLD group and treatments preference order suggested by the COPD‐CDSS. However, no preference ordering is maintained when launching hook ‘copd‐careplan‐review’ via button ‘Personalised care plan’, that is, ticked checkboxes linked to field ‘Suggested treatments’ are considered equally preferred. This design choice was made by Heliant to simplify user interaction. Consequently, preferences and goals were left out of the COPD‐CDSS evaluation.

**FIGURE 6:**
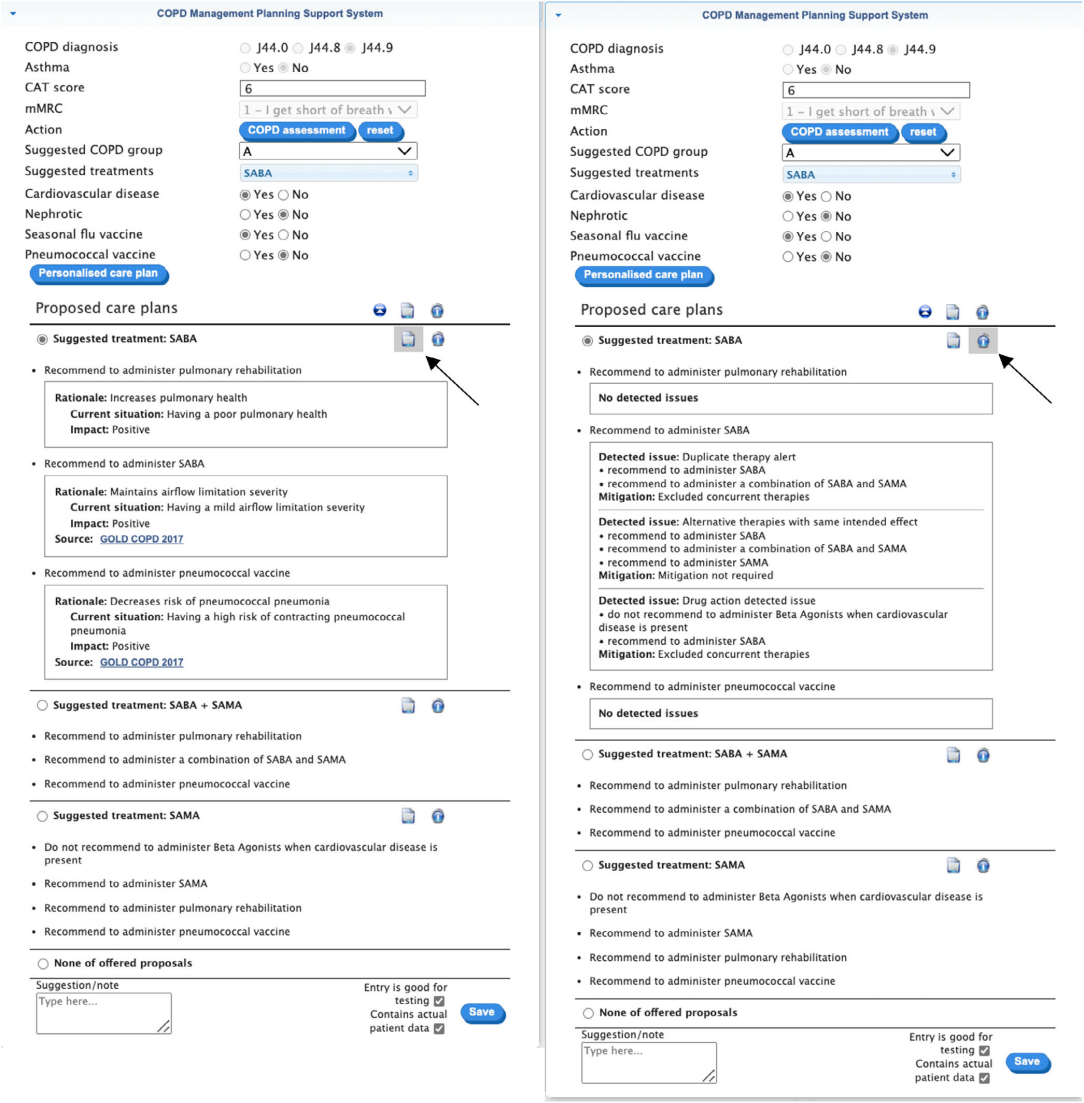
COPD treatment planning response by COPD‐CDSS for the patient with EHR and COPD severity assessment displayed in Figure 5. Results are split into two tabs, presented here as one image for the sake of readability. The left tab displays the rationale behind each proposed recommendation; the right tab displays mitigation results for the identified potential interactions among proposed recommendations. A black arrow was added to the image to indicate the button that displays each tab when clicked.

Figure 6 shows the selection made by the pulmonologist when triggering hook ‘copd‐careplan‐review’ and the resulting EHR‐integrated card. The COPD‐CDS response proposed three personalized care plans, the top one has SABA as COPD treatment, which was the sole selection made by the pulmonologist in field ‘Suggested treatments’. The additional proposals provide alternative COPD treatments for the user‐selected COPD group as taken from the ‘copd‐assess’ response. Additional proposals are added due to detected contradictory recommendations involving beta‐agonists (see Figure 1). As a beta‐agonist, SABA is not recommended for susceptible COPD patients with comorbid CVD. Application of the SABA‐based care plan does not resolve the conflict, some of the other proposals do, so the drug‐disease conflict warning was included as an explanation in the proposal with no beta‐agonists. In addition, there are recommendations for pulmonary rehabilitation therapy and for the pneumococcal vaccination. The former is common to all COPD patients, the latter is due to the patient's age (66). The rationale behind each proposed recommendation (Figure 6, right tab) is depicted in a structured form, generated from the encoded terms provided by the explainability algorithm, part of the conflict resolution engine. This design choice was chosen over the generated (English) textual representation (eg, Table 2, column Explanation) as it enhances scalability and simplifies translation: encoded TMR terms support formal interpretation (eg, finding SNOMED CT representatives, however mapping back TMR‐based knowledge to SNOMED CT was not part of the evaluation). The right tab in Figure 6 enumerates potential interactions found in the SABA‐driven care plan (depicted in Figure 1) and how they were resolved (here, by excluding interacting treatments from this proposed care plan). The text utilises a mix of FHIR detectedIssue resources and ROAD2H‐defined nomenclature to describe conflicts and their resolutions.

## 4 EVALUATION

### 4.1 Agreement with COPD‐CDSS results

Data derived from 99 EHRs were returned by the events log of both the COPD‐CDSS and Heliant's EHR system when each pulmonologist assessed their 20 cases once. When verifying each EHR, we found 65 cases where entry data did not match that of the provided case vignettes. Many of these modifications were dynamically inserted by pulmonologists at evaluation using the provided GUI, so we deduced they chose to challenge the COPD‐CDSS outside the boundaries of the provided cases. However, as we focus on the pulmonologist behaviour for each EHR and associated CDS service, these discrepancies do not affect the overall evaluation. We found two Serbian mistranslations of clinical recommendations that may have left clinicians with a misleading understanding (see Appendix B.2, Table 8).

Reported results matched proposed COPD‐CDSS outcomes with pulmonologists' decisions for the same cases. Pulmonologists agreed 97% of the time with the GOLD group assigned by COPD‐CDSS to each EHR. The remaining 3% found a discrepancy between the use case and the EHR‐stored data. Personalised COPD‐related therapies were also suggested, and prioritized, for each case. Our analysis indicated pulmonologists corroborated the most favoured therapy for 31.3% of the cases whereas second, third, and fourth preferences had 33.3, 24.2, and 9.1 selection rates, respectively. Rejection of all suggested therapies was 2% and accordant with the previous 3% of discrepancy.

Next, the COPD‐CDSS presented one or more alternative care plan proposals for each case, based on the pulmonologist's selected GOLD group and corresponding therapy. Each proposal incorporated personalised recommendations and warnings. Pulmonologists rejected all alternative proposals in the same selection for 1% of the cases while at least one of the proposals was deemed suitable for 71.7% of the cases. A satisfactory proposal was derived by the pulmonologists for the remaining 27.3% of the cases by de‐selecting a pair of mistranslated recommendations (English to Serbian) from some results. Incorporating recommendations from alternative proposals was allowed; however, none of the pulmonologists deemed necessary to augment their selected proposal for each case. Interestingly, for 72% of the cases, clinicians selected the topmost displayed proposal from the unordered collection presented on the GUI.

### 4.2 Qualitative analysis

Following use of the COPD‐CDSS integrated with Heliant's EHR and the 20 vignettes, five pulmonologists were invited to take part in individual interviews and responses were documented by the researcher. A structured protocol was used as a guide to explore perceptions in using the COPD‐CDSS with case vignettes. For a full list of evaluation questions and answers, see Appendix B.3. The following themes were developed from examining all clinicians' responses:

#### 4.2.1 Treatment and management options

All five pulmonologists agreed the CDS offered a wide range of treatment options. One respondent suggested the CDS offered appropriate treatment choices including alternative therapeutic options.

*Offered options for medication, based on the entered parameters on severity and discomfort of disease. Also, the idea of non‐medical recommendations. (Doctor 2)*.

One respondent suggested the drug therapies presented could be more precise.

*The data must be more precise. E.g., drugs from the group of beta‐agonists are divided into SABA and LABA, and it must be clear to which therapeutic option the data refers to… (Doctor 1)*.

#### 4.2.2 Functionality and usability

All five clinicians agreed there were no features of the COPD‐CDSS they found unhelpful or ‘least useful’.

The CDS was positively regarded as a *“Clear and concise software” (Doctor 3)*. Although technical issues related to data entry were raised, one respondent stated these were easily resolved.

*… Minor technical problems (that) were easily resolved in consultations and with the support of the IT specialist (Doctor 3)*.

One respondent suggested future development of the CDS should aim to be more streamlined.

*“The goal of every system is simplicity, speed, and efficiency, with as few unnecessary pop‐ups and additional questions as possible. (Doctor 5)*.

One way of using the CDS efficiently is integration into existing systems to reduce workload.

*Integration with the existing hospital information system, so that all required data for the patient is already present in the EHR system … Avoiding the unnecessary entering of the same data is a waste of time (Doctor 2)*.

#### 4.2.3 Patient monitoring and data capture

The CDS was thought to be useful for continual health monitoring purposes.

*In situations when the patient comes regularly for examinations, for better monitoring (Doctor 4)*.

However, most respondents suggested a wider range of patient data should be captured including co‐morbidities, diagnostic investigations, and other therapeutic options.

*As much patient data as possible should be entered into the system (from the EHR) (Doctor 4)*.

*Not enough options for entering the data on associated diseases of importance (Doctor 1)*.

*Functions of including other aspects (findings) such as spirometry, lab tests, X‐rays (Doctor 3)*.

*No therapeutic option for ICS in deciding on a therapeutic option when entering data for patients who also have asthma (Doctor 3)*.

#### 4.2.4 Additional uses of the CDS

One respondent thought the CDS would be an effective companion tool for less experienced clinicians.

*The system is a very good idea and a kind of guideline for doctors with little clinical experience at the very beginning of independent management of patients, and then their outpatient examinations. (Doctor 5)*.

Respondents also said that CDS could be used to aid decisions for other diseases.

*It can serve as an advising tool and as a reminder of other guidelines if they exist in other diseases (Doctor 2)*.

*It would not be bad to expand the field of action to other specialties (gastroenterology, endocrinology, cardiology…) and thus help in deciding the patient with co‐morbidities initially during outpatient consultations. (Doctor 5)*.

## 5 DISCUSSION

We successfully extended the TMR ontological framework for expressing guideline recommendations to provide individual patient‐based reasoning and explanation via argumentation. In addition, a CDS microservices architecture based on open standards (SNOMED CT, HL7 FHIR and CDS Hooks) was used to embed the resulting TMR‐based CDS framework into a commercial EHR platform. Although the system can manage more than two interactions at a time, our evaluation was limited to interactions arising from a single guideline because of the nature of the clinical use case. In clinical practice patients with co‐morbidities will have multiple applicable guidelines, potentially increasing the complexity of interactions. Further development of the TMR‐based CDS framework to cover multiple guidelines is needed to fully evaluate the robustness of our approach. Furthermore, due to the COVID‐19 pandemic, the evaluation in Serbia was delayed for 12 months and was eventually only able to go ahead with five pulmonologists and 20 clinical vignettes rather than live in a COPD clinic as planned. The EHR vendor also introduced several restrictions in that some of the clinical vignette data had to be entered by the clinician at the start, rather than being already present in the EHR, and the ability to order recommendations by prior patient preference and overall clinical goals, for example, cost/effectiveness, was omitted. Many of the comments in the qualitative analysis reflect these limitations rather than inherent issues with the approach.

In the practical application of the approach, we note several limitations. First, the representation of the GOLD statements in TMR is time‐consuming, requiring input from experienced clinicians and knowledge engineers. This problem is common to all knowledge representation approaches including PROFORMA,^25^ Arden Syntax,^26^ and CQL.^27^ However, as TMR relationships are defined using the semantic web, this may offer a potential route for semi‐automation of the guideline representation process. A combination of natural language processing and expression of found concepts and relationships as knowledge graphs, constrained by the TMR ontology, would be the next research step. The current version of TMR was suitable to represent the cyclic workflow style of managing a chronic condition like stable COPD; however, more complex scenarios will require for TMR to handle temporal reasoning, drug dosing and guideline flow control. Future research will explore these topics. In terms of integration with EHRs, FHIR is being increasingly adopted and CDS Hooks is a non‐proprietary standard, so the approach we took with Heliant should be widely replicable in other LMIC where open‐source EHRs based on standards are of value. Guidelines represented in TMR are also a shareable open resource, with potential for local adaptation and optimisation in a transparent way. Although existing syntaxes such as CQL have much greater maturity than TMR, TMR being an ontological approach is much more extensible (as in our addition of argumentation) and a better fit with advances in ontology‐based knowledge extraction methods such as neural‐symbolic reasoning.^28^

Arguably, the ABA+G explanations delineated above do not make use of the full spectrum of explanation techniques available in argumentation. Nevertheless, together with the explainable nature of argumentation, they are a steppingstone in meeting explainability guidelines for the deployment of AI‐assisted systems produced by the UK Information Commissioner's Office and the Turing Institute. These explanations indicate the reasoning underlying the recommendations in the spirit of Explainable AI methods drawn from argumentative abstractions. As future work, we would explore whether interactive forms of explanations, as in asking questions, naturally supported by argumentation, could be an alternative approach to providing explanation in our context.^29^

Microservices are independently manageable services, creating applications that improve scalability, are more resilient and have better fault isolation. For instance, a strategy to extend the reach of the COPD‐CDSS to multiple Serbian healthcare institutions would entail the distribution of incoming CDS requests among multiple instances of each microservice in the TMR‐based architecture. Then, a load balancer server would act as proxy between the clients and the CDS hooks manager service instances, distributing CDS requests according to an algorithm. Similarly for the interaction among the components of the CGs enactment engine. Furthermore, the enhancement of the generic CDS system to manage other chronic diseases such as diabetes or cardiovascular disease could be achieved by formalising the respective guideline(s) using TMR and by deploying a separate TMR‐based CDS services manager microservice linked to the other existing CGs enactment engine components. Then, new CDS services would be registered by designing both dedicated CDS hooks and their corresponding JSON‐based documents (the latter added to the CDS hooks manager microservice database). In this context, the COPD‐CDSS would not be affected by the deployment or execution of the newly registered CDS services, for instance if some complex functionality applicable to the parameters in the corresponding JSON‐based document fails (see point number 2 in Figure 3).

The ROAD2H approach to embedding computable guidelines into decision support tools is very much aligned with the WHO SMART guideline initiative which aims to support translation of existing knowledge into computable guidelines using Digital Adaptation Kits (DAKs). The TMR model provides a blueprint for implementing the business processes & workflows and decision support elements of a DAK, and we are currently looking into developing ROAD2H‐based DAKs for multimorbidity scenarios.

## 6 CONCLUSION

We approached the problem of supplying explainable AI in the form of a CDSS for guidelines by representing guideline statements and an assessment of the patient's disease stage using an existing ontological model of guideline recommendations, the TMR model, and building argumentation as an additional reasoning layer on top of it. On the example of COPD, we proved that it is possible to transact guideline statements, recommendations and contraindications using this approach and to implement them using a microservice model incorporating widely used standards (SNOMED CT, FHIR API and CDS Hooks). In addition, the system was implemented in integration with a commercial EHR widely used in Serbia and other West Balkans LMICs. A mixed‐method evaluation using vignettes also showed high agreement with pulmonologists and favourable views on the implementation and the potential of such systems. Thus, the strategy of providing standard‐based, explainable, and reproducible CDS that integrate in real‐time with clinical systems shows promise and should be explored further.

## AUTHOR CONTRIBUTIONS

**Jesús Domínguez**: Software engineering; computing design; writing; final draft approval. **Denys Prociuk**: Software engineering; data collection; analysis; writing; final draft approval. **Branko Marović**: Conceptualisation and funding; software engineering; computing design; data collection; analysis; project administration and leadership; writing; final draft approval. **Kristijonas Čyras**: Final draft approval. **Oana Cocarascu**: Software engineering; computing design; analysis; final draft approval. **Francis Ruiz**: Conceptualisation and funding; analysis; project administration and leadership; writing; final draft approval. **Ella Mi**: Clinical feedback; data collection; analysis; writing; final draft approval. **Emma Mi**: Clinical feedback; data collection; analysis; writing; final draft approval. **Christian Ramtale**: Data collection; analysis; writing; final draft approval. **Antonio Rago**: Software engineering; computing design; analysis; final draft approval. **Ara Darzi**: Conceptualisation and funding; clinical feedback; project administration and leadership; writing; final draft approval. **Francesca Toni**: Conceptualisation and funding; software engineering; computing design; project administration and leadership; writing; final draft approval. **Vasa Curcin**: Conceptualisation and funding; software engineering; computing design; project administration and leadership; writing; final draft approval. **Brendan Delaney**: Conceptualisation and funding; software engineering; clinical feedback; analysis; project administration and leadership; writing, final draft approval.

## FUNDING INFORMATION

The study was funded by the Engineering and Physical Sciences Research Council Global Challenges Research Fund grant ROAD2H: Resource Optimisation, Argumentation, Decision Support, and Knowledge Transfer to Create Value via Learning Health Systems (EP/P029558/1), the UK Research and Innovation, and Health Data Research UK. The authors gratefully acknowledge infrastructure support from the National Institute for Health and Care Research (NIHR) Imperial Patient Safety Translational Research Centre, the NIHR Imperial Biomedical Research Centre.

## CONFLICT OF INTEREST STATEMENT

The authors declare that they have no financial or non‐financial competing interests.

## Supporting information


**Data S1.** Supporting Information.

**Appendix S1.** Supporting Information.

## Data Availability

All data from the evaluation are provided in Appendix B. All code and details of installation are available as described in Appendix C: Technical documentation.

## References

[1] National Institute for Health and Care Excellence (NICE) . [cited 2022 Mar 8]. https://www.nice.org.uk/

[2] WHO . Multimorbidity. Technical Series on Safer Primary Care. Geneva: World Health Organisation; 2016.

[3] Zamborlini V , da Silveira M , Pruski C , et al. Analyzing interactions on combining multiple clinical guidelines. Artif Intell Med. 2017;1(81):78‐93. 10.1016/j.artmed.2017.03.012 28410780

[4] Riaño D , Ortega W . Computer technologies to integrate medical treatments to manage multimorbidity. J Biomed Inform. 2017;75:1‐13. [cited 2020 Mar 8] https://linkinghub.elsevier.com/retrieve/pii/S153204641730206X 28942139 10.1016/j.jbi.2017.09.009

[5] Fraccaro P , Casteleiro MA , Ainsworth J , Buchan I . Adoption of clinical decision support in multimorbidity: a systematic review. JMIR Med Inform. 2015;3(1):e4 [cited 2020 Mar 4] http://medinform.jmir.org/2015/1/e4/ 25785897 10.2196/medinform.3503 PMC4318680

[6] Peleg M . Computer‐interpretable clinical guidelines: a methodological review. J Biomed Inform. 2013;46(4):744‐763. [cited 2021 Nov 18] http://www.ncbi.nlm.nih.gov/pubmed/23806274 23806274 10.1016/j.jbi.2013.06.009

[7] Ancker JS , Edwards A , Nosal S , Hauser D , Mauer E , Kaushal R . Effects of workload, work complexity, and repeated alerts on alert fatigue in a clinical decision support system. BMC Med Inform Decis Mak. 2017;17(1):36. [cited 2022 Oct 5]. doi:10.1186/s12911-017-0430-8 28395667 PMC5387195

[8] Walsh K , Wroe C . Mobilising computable biomedical knowledge: challenges for clinical decision support from a medical knowledge provider. BMJ Health Care Inform. 2020;27:e100121. 10.1136/bmjhci-2019-100121 PMC7388874 32723850

[9] Asogwa OA , Boateng D , Marzà‐Florensa A , et al. Multimorbidity of non‐communicable diseases in low‐income and middle‐income countries: a systematic review and meta‐analysis. BMJ Open. 2022;12(1):e049133 [cited 2022 Sep 15] https://bmjopen.bmj.com/content/12/1/e049133 10.1136/bmjopen-2021-049133 PMC8785179 35063955

[10] HL7 International . CDS Hooks. [cited 2020 Jan 1]. https://cds-hooks.org/

[11] Greenes RA , Bates DW , Kawamoto K , Middleton B , Osheroff J , Shahar Y . Clinical decision support models and frameworks: seeking to address research issues underlying implementation successes and failures. J Biomed Inform. 2018;78:134‐143. [cited 2022 Jan 20] http://www.ncbi.nlm.nih.gov/pubmed/29246790 29246790 10.1016/j.jbi.2017.12.005

[12] Mercier H , Sperber D . Why do humans reason? Arguments for an argumentative theory. Behav Brain Sci. 2011;34(2):57‐74. 21447233 10.1017/S0140525X10000968

[13] Čyras K , Oliveira T , Karamlou A , Toni F . Assumption‐based argumentation with preferences and goals for patient‐centric reasoning with interacting clinical guidelines. Argument Comput. 2021;12(2):149‐189. [cited 2021 Jul 15]. doi:10.3233/AAC-200523

[14] GOLD . GOLD 2017 Global Strategy for the Diagnosis, Management and Prevention of COPD. Global Initiative for Chronic Obstructive Lung Disease; [cited 2023 June 6] https://goldcopd.org/wp-content/uplolads/2017/02/wms-GOLD-2017-FINAL.pdf 2017.

[15] Heliant Health Healthcare Information system. [cited 2021 Oct 11]. https://heliant.rs/?lang=en

[16] Zamborlini V , Hoekstra R , da Silveira M , Pruski C , ten Teije A , van Harmelen F . Inferring recommendation interactions in clinical guidelines. Schlobach S, Janowicz K, Schlobach S, Janowicz K. Semant Web. 2016 [cited 2022 Feb 11];7(4):421‐446. doi:10.3233/SW-150212

[17] Prud'hommeaux E , Seaborne A . SPARQL 1.1. Query Language for RDF. 2007 [cited 2022 Feb 21]. https://www.w3.org/TR/rdf-sparql-query/

[18] Hunter A , Williams M . Aggregating evidence about the positive and negative effects of treatments. Artif Intell Med. 2012;56(3):173‐190. 23178172 10.1016/j.artmed.2012.09.004

[19] Moulin B , Irandoust H , Bélanger M , Desbordes G . Explanation and argumentation capabilities: towards the creation of more persuasive agents. Artif Intell Rev. 2002;17(3):169‐222.

[20] Schulz C , Toni F . Justifying answer sets using argumentation. Theory Pract Logic Prog. 2016;16(1):59‐110.

[21] García AJ , Chesñevar CI , Rotstein ND , Simari GR . Formalizing dialectical explanation support for argument‐based reasoning in knowledge‐based systems. Expert Syst Appl. 2013;40(8):3233‐3247.

[22] Čyras K , Birch D , Guo Y , et al. Explanations by arbitrated argumentative dispute. Expert Syst Appl. 2019;127:141‐156. https://linkinghub.elsevier.com/retrieve/pii/S0957417419301654

[23] Cyras K , Oliveira T . Argumentation for reasoning with conflicting clinical guidelines and preferences. Principles of Knowledge Representation and Reasoning: Proceedings of the 16th International Conference, KR 2018; AAAI Press, Palo Alto, CA, 2018.

[24] Chapman M , Curcin V . A microservice architecture for the design of computer‐interpretable guideline processing tools. IEEE EUROCON 2019—18th International Conference on Smart Technologies. IEEE; 2019 [cited 2020 Mar 6]:1‐6 https://ieeexplore.ieee.org/document/8861830/

[25] Fox J , Johns N , Lyons C , Rahmanzadeh A , Thomson R , Wilson P . PROforma: a general technology for clinical decision support systems. Comput Methods Prog Biomed. 1997 Sep;54(1–2):59‐67. https://linkinghub.elsevier.com/retrieve/pii/S0169260797000345 10.1016/s0169-2607(97)00034-5 9290920

[26] Hripcsak G , Ludemann P , Pryor TA , Wigertz OB , Clayton PD . Rationale for the Arden syntax. Comput Biomed Res. 1994;27(4):291‐324. https://linkinghub.elsevier.com/retrieve/pii/S0010480984710238 7956129 10.1006/cbmr.1994.1023

[27] Arasu A , Babu S , Widom J . The CQL continuous query language: semantic foundations and query execution. VLDB J. 2006;15(2):121‐142.

[28] Zhang J , Chen B , Zhang L , Ke X , Ding H . Neural, Symbolic and Neural‐Symbolic Reasoning on Knowledge Graphs. AI Open 2:14‐35; 2021.

[29] Rago A , Cocarascu O , Bechlivanidis C , Lagnado D , Toni F . Argumentative explanations for interactive recommendations. Artif Intell. 2021;296:103506.

